# A giant retropharyngeal space lipoma: a case report and review of the literature

**DOI:** 10.3389/fsurg.2026.1783809

**Published:** 2026-06-24

**Authors:** Takashi Koike, Ryo Takagi, Kosei Mori, Kengo Kanai, Yoshihiro Watanabe, Mitsuhiro Okano, Yoshihiro Noguchi, Yuichiro Hayashi, Mina Komuta, Yorihisa Imanishi

**Affiliations:** 1 Department of Otorhinolaryngology–Head and Neck Surgery, International University of Health and Welfare, School of Medicine, Narita Hospital, Narita, Japan; 2 Department of Pathology, International University of Health and Welfare, School of Medicine, Narita Hospital, Narita, Japan

**Keywords:** case report, dysphagia, giant, nerve integrity monitor, retropharyngeal lipoma, tracheostomy, transcervical resection

## Abstract

**Background:**

Lipoma is the most common benign mesenchymal tumor; however, its occurrence in the retropharyngeal space (retropharyngeal lipoma [RPL]) is exceedingly rare. Due to the softness and distensibility of the retropharyngeal space, RPLs can enlarge significantly without causing any symptoms, resulting in wide variability in tumor size at diagnosis. Although tumor size inevitably influences symptom burden and treatment strategies, no review has focused specifically on “giant” RPLs. Herein, we report a case of exceptionally large RPL and review previously reported giant cases with a maximum diameter of ≥ 10 cm.

**Case presentation:**

A 55-year-old Japanese male presented with mild dyspnea, progressive dysphagia, anterior neck swelling, and long-standing worsening snoring. Endoscopy demonstrated submucosal bulging of the posterior pharyngeal wall. Computed tomography revealed a well-circumscribed, non-enhanced, fat density mass measuring 140 × 110 mm in the retropharyngeal space, extending from the level of the uvula to the lower pole of the thyroid and displacing the pharyngolarynx, trachea, esophagus, thyroid gland, and common carotid arteries. An imaging diagnosis of RPL was confirmed by magnetic resonance imaging. Given the enormous size and anticipated risk of postoperative airway compromise, transcervical resection with prophylactic tracheostomy was performed using a nerve integrity monitor (NIM). The excised tumor measured 145 × 120 × 40 mm and weighed 240 g. Histopathological findings further corroborated the diagnosis of lipoma. Postoperatively, oral intake gradually normalized, and all preoperative symptoms fully resolved within two months.

**Conclusions:**

This case highlights the need to recognize giant RPL as one of the differential diagnoses in patients with gradually worsening dysphagia and the efficacy of its surgical treatment. A literature review of giant RPL cases suggested an even higher predominance in males, a greater symptom multiplicity, and a more frequent use of the transcervical approach. NIM was considered useful in this case as an adjunct for locating the recurrent laryngeal nerve, while the necessity of prophylactic tracheostomy and postoperative tube feeding should be assessed individually.

## 1 Introduction

Lipomas are the most common benign mesenchymal tumors and may arise in any anatomical location (1–3). Approximately 13% of lipomas are located in the head and neck region, most commonly in the subcutaneous space of the posterior neck triangle (1–5). However, lipomas may also arise in the retropharyngeal space, where they are considered exceedingly rare (2–5).

According to a few recent reviews of accumulated case reports on retropharyngeal lipoma (RPL) (4, 5), this lesion exhibits a higher prevalence in males and may present with a wide range of clinical symptoms attributed to pressure effects on adjacent structures, with dysphagia being the most common, followed by snoring, dyspnea, dysphonia, and obstructive sleep apnea. Computed tomography (CT) is sufficient for the imaging diagnosis of RPL, typically displaying a well-circumscribed, non-enhancing, homogeneous lesion with fat density. Furthermore, magnetic resonance imaging (MRI) provides more detailed information regarding the relationship of the lesion with surrounding structures, along with characterization of the lipoma signal, which is attenuated by using a fat-suppression sequence (4, 5). Most patients with RPL undergo surgical excision via either the transcervical or transoral approach (4–6). Although the largest-ever review of RPL case reports by Chrysovitsiotis (4) was thorough and comprehensive, the extremely broad reporting period warrants the need for a more up-to-date review focused on recent medical standards.

RPLs potentially attain a considerable size without causing any symptoms due to their softness and elasticity, as well as the distensibility of the retropharyngeal space. However, since the extent of asymptomatic growth varies among individuals, the size of the RPLs demonstrates a remarkably wide range, as noted in previous review articles (4, 5). Although the size of an RPL is expected to significantly influence both symptom severity and the choice of surgical approach, no review has focused specifically on “giant” RPLs.

Here, we report a patient with an extraordinarily large RPL presenting with multiple symptoms who underwent surgical excision via the transcervical approach with preventive tracheostomy. Moreover, the study reviews previous case reports of RPL with particular emphasis on cases of giant size.

## 2 Case presentation

### 2.1 Preoperative assessment

**Figure 1:**
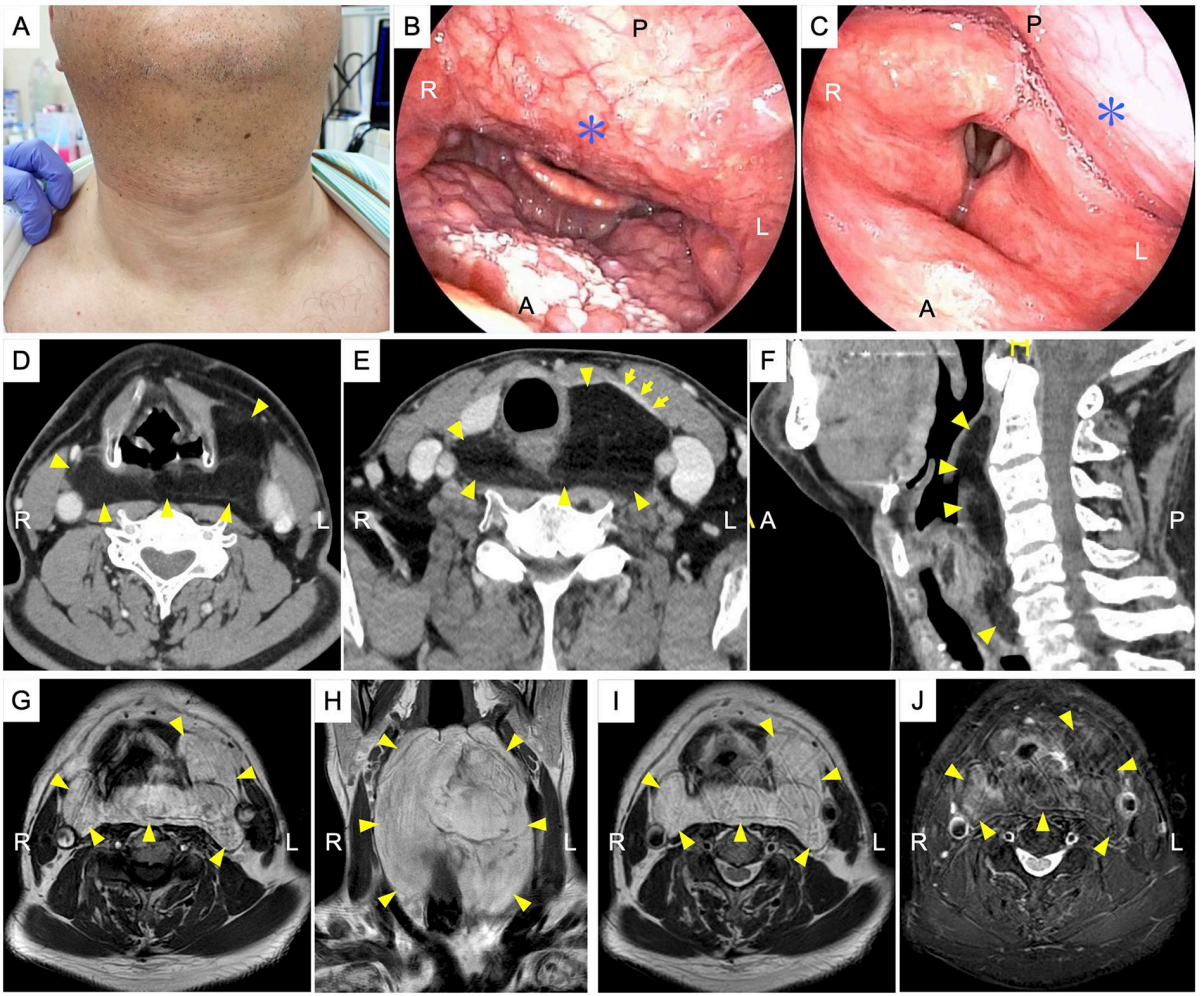
Physical examination, endoscopic evaluation, and imaging findings on computed tomography (CT) and magnetic resonance imaging (MRI). **(A)** Physical examination demonstrates diffuse subcutaneous swelling of the neck. **(B,C)** Flexible endoscopic findings revealing submucosal bulging (*) of the posterior pharyngeal wall from the oropharynx **(B)** to the hypopharynx **(C)**. **(D–F)** CT images displaying a well-circumscribed, non-enhanced, fat-density mass (arrowheads) in the retropharyngeal space, extending vertically from the level of the uvula to the lower pole of the thyroid gland **(F)**, displacing the pharyngolarynx, trachea, esophagus, and thyroid gland, particularly the left lobe (**(E)**, arrows) anteriorly, and the common carotid arteries bilaterally **(D,E)**. **(G–J)** MRI displaying a lesion (arrowheads) with thin septations, exhibiting hyperintense signal on both T1-weighted **(G,H)** and T2-weighted **(I)** images, with obvious signal suppression on fat-saturated sequences **(J)**, consistent with the diagnosis of lipoma. (R, right; L, left; A, anterior; P, posterior).

A 55-year-old Japanese male presented to a local clinic with mild dyspnea along with sputum retention for two months, as well as progressively worsening difficulty in swallowing and anterior neck swelling for the past month. Since plain CT revealed a mass in the retropharyngeal space, the patient was referred to our hospital for further evaluation. His medical history was unremarkable except for moderate obesity (height: 170.5 cm; weight: 87 kg; body mass index: 30) accompanied by longstanding snoring, which appeared to have gradually worsened over time. Physical examination revealed diffuse subcutaneous swelling of the neck upon palpation (Figure 1A). Flexible endoscopy demonstrated submucosal bulging of the posterior pharyngeal wall extending from the oropharynx to the hypopharynx (Figures 1B,C), together with epiglottic fluttering during inhalation due to its contact with the posterior pharyngeal wall. No laryngeal paralysis, including vocal cord immobility, was observed. Laboratory test showed elevated triglyceride and low-density lipoprotein cholesterol levels, whereas other hematologic and biochemical parameters remained within reference limits.

Contrast-enhanced CT demonstrated a well-circumscribed, non-enhanced, fat-density mass measuring approximately 140 mm transversely and 110 mm craniocaudally in the retropharyngeal space. The lesion extended superiorly to a level of the uvula and inferiorly to the lower pole of the thyroid, displacing the pharyngolarynx, trachea, esophagus, thyroid gland anteriorly, and the common carotid arteries bilaterally (Figures 1D–F). Notably, the left thyroid lobe appeared markedly thinned and flattened due to tumor compression (Figure 1E). MRI revealed an encapsulated mass with thin septations, exhibiting a hyperintense signal on both T1-weighted (Figures 1G,H) and T2-weighted (Figure 1I) images, with obvious signal suppression on fat-saturated sequences (Figure 1J). These imaging findings established the diagnosis of giant RPL. Given the tumor's considerable size and the anticipated risk of postoperative airway compromise, surgical resection via an external incision with prophylactic tracheostomy was planned.

### 2.2 Surgical findings

**Figure 2:**
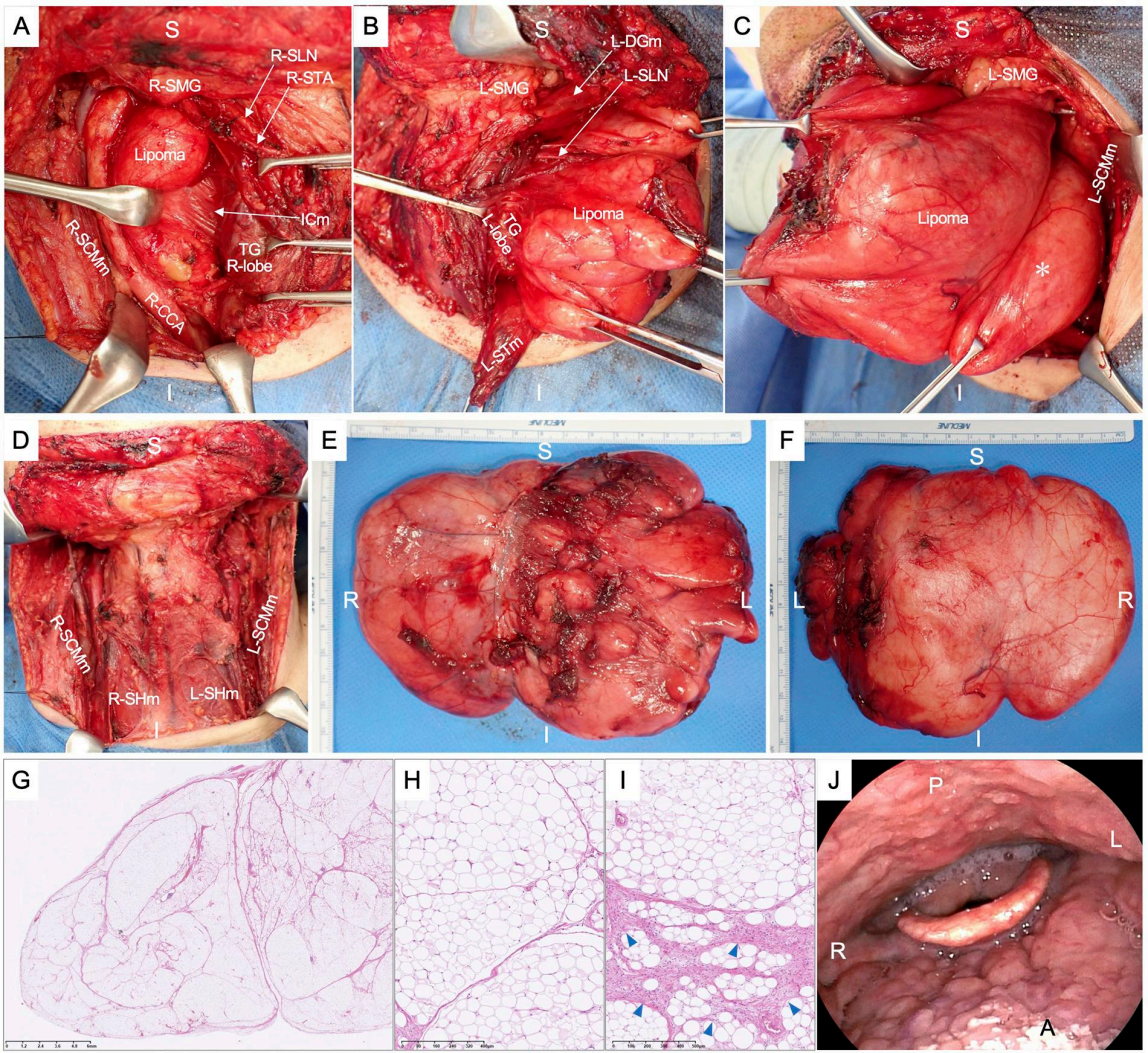
Operative, histopathological, and postoperative endoscopic findings. **(A–D)** Intraoperative views. **(A)** On the right side, the tumor is dissected from the carotid sheath, right thyroid lobe, trachea, inferior constrictor, and esophageal muscles, leaving the internal branch of the right superior laryngeal nerve (SLN) and superior thyroid artery (STA) intact. **(B)** On the left side, the tumor is dissected from the surrounding tissues, as on the right side. Despite marked thinning due to posterior tumor compression, the left thyroid lobe was carefully preserved along with the internal branch of the left SLN, whereas the severely narrowed left STA was resected. **(C)** Following complete dissection from the prevertebral fascia, the right portion of the lipoma was pulled out toward the left (*) using a pull-through maneuver between the pharyngoesophagus and the prevertebral fascia. **(D)** Anterior view of the neck after tumor extirpation. **(E,F)** Gross appearance of the excised tumor displaying the ventral **(E)** and dorsal **(F)** surfaces. The tumor measured approximately 145, 120, and 40 mm in the horizontal, vertical, and anteroposterior diameters, respectively, and weighed 240 g. **(G–I)** Histopathological findings: hematoxylin and eosin-stained sections revealed typical mature adipocytes **(H)** forming a lobular architecture **(G)**, with only a small portion of fibrous stroma containing lymphocyte-predominant infiltration (**(I)**, arrowheads). No malignant features were observed, confirming the diagnosis of a lipoma. **(J)** Postoperative endoscopic findings: Two months postoperatively, the disappearance of the posterior pharyngeal wall bulging was confirmed. (S, superior; I, inferior; R, right; L, left; A, anterior; P, posterior; CCA, common carotid artery; DGm, digastric muscle; ICm, inferior constrictor muscle; TG L-lobe, left lobe of thyroid gland; TG R-lobe, right lobe of thyroid gland; SCMm, sternocleidomastoid muscle; SHm, sternohyoid muscle; SLN, superior laryngeal nerve; SMG, submandibular gland; STA, superior thyroid artery; STm, sternothyroid muscle).

Surgery was performed under general anesthesia using a nerve integrity monitor (NIM) in consideration of the potential risk of recurrent laryngeal nerve (RLN) injury. Following a U-shaped cervical skin incision and elevation of the platysmal-cutaneous flaps, tumor resection was initiated on the right (smaller) side. The lesion was carefully dissected from the carotid sheath, right thyroid lobe, trachea, inferior constrictor, and esophagus while preserving its thin capsule, as well as the internal branch of the right superior laryngeal nerve (SLN) and superior thyroid artery (STA) (Figure 2A). The absence of marked intertissue adhesion allowed for the right RLN preservation without exposure. After dissection of the dorsal tumor surface from the prevertebral fascia beyond the midline, the procedure proceeded to the left side. Although the dorsal surface of the left thyroid lobe was firmly adherent to the tumor, the left lobe and internal branch of the left SLN were carefully preserved through meticulous dissection; however, the markedly narrowed left STA was sacrificed (Figure 2B). Similar to the right side, the tumor was dissected from the carotid sheath, trachea, inferior constrictor, and esophagus on the left side, with preservation of the left RLN without direct exposure. The functional integrity of both RLNs was confirmed by bilateral vagus nerve stimulation using NIM. Following complete dissection from the prevertebral fascia, the right side of the lesion was pulled out toward the left using a pull-through maneuver between the pharyngoesophagus and the prevertebral fascia (Figure 2C). Eventually, the tumor was extirpated *en bloc* without causing any additional damage to the surrounding tissues (Figure 2D), followed by preventive tracheostomy. The excised specimen measured approximately 145 × 120 × 40 mm (horizontal × vertical × anteroposterior) and weighed 240 g (Figures 2E,F).

Histopathological examination revealed proliferation of typical mature adipocytes exhibiting lobular growth patterns (Figures 2G,H), with only a small portion of fibrous stroma containing lymphocyte-predominant infiltration, reflecting chronic inflammation (Figure 2I). No significant cytologic atypia, lipoblasts, or fibrous septa with atypical stromal cells were observed. In addition, there were no morphological findings suggestive of atypical lipomatous tumor or well-differentiated liposarcoma, such as nuclear atypia or hyperchromasia of stromal cells, confirming the diagnosis of lipoma.

### 2.3 Postoperative course

Intravenous corticosteroids were administered for postoperative pharyngeal and laryngeal edema, which gradually subsided over the course of one week. Transient subcutaneous emphysema secondary to tracheostomy resolved spontaneously. Neither recurrent laryngeal nerve palsy nor voice dysfunction was observed postoperatively. The patient resumed oral intake of a liquid diet on postoperative day (POD) 2 and regained a normal diet by POD 14. The drainage tubes were removed on POD 5, and the tracheostoma was closed on POD 7.

At the two-month postoperative follow-up, all preoperative symptoms, including mild dyspnea and swallowing difficulty, had fully resolved, except for longstanding snoring. Although the snoring was partly attributable to obesity, it also improved to a certain extent. The posterior pharyngeal wall bulging also disappeared, thereby restoring the narrowed anterior-posterior patency of the upper airway (Figure 2J). The patient was recurrence-free after one year of follow-up.

## 3 Discussion

According to previous comprehensive reviews of the RPL case reports, tumor size varied considerably, with maximum diameters ranging from 3.0 to 19.0 cm and an average of 8.3–9.0 cm (4, 5). In these reviews, the first study (*n* = 79) reported 37 cases (46.9%) with a maximum diameter <10 cm, 25 cases (31.6%) ≥ 10 cm, and 17 cases (21.5%) with missing information (4), while the second study (*n* = 27) reported 15 cases (55.6%) < 10 cm, six cases (22.2%) ≥ 10 cm, and six cases (22.2%) with missing information (5). Furthermore, because more than half of the cases in the first review were reported prior to 2000, with the earliest publication dating back to the 19th century, contemporary clinical practice may not be optimally reflected.

Hence, to better understand the clinical characteristics and current management of giant RPL, we conducted a literature review exclusively of cases reported after 2000, defining giant RPL as lesions with a maximum diameter of at least 10 cm. A database search was performed using PubMed, Embase, Scopus, Web of Science, and Google Scholar, with the search strategy including the following terms: (“retropharyngeal lipoma” OR “retropharyngeal space lipoma” OR (“lipoma” AND “retropharyngeal”)) AND (“case report” OR “case reports”). Articles that contained at least an English abstract and were published by December 2025 were included. After removal of duplicates through screening titles and abstracts, full-text papers were assessed according to predefined inclusion criteria (articles describing individual cases of giant RPL) and exclusion criteria (non-English articles, review articles without individual patient data, and irrelevant conditions). A total of 16 cases were ultimately identified; Table 1 presents their key clinical information, including the present case (5, 7–21). Furthermore, the epidemiological, diagnostic, and treatment-related data are summarized in Table 2 (5, 7–21). The age ranged from 13 to 75 years, with a median of 48 years and an average of 50 ± 17 years. With respect to age distribution, the majority of patients were 40 years or older, with those aged 40–59 accounting for nearly half (47.1%). The sex distribution demonstrated male predominance, with a male-to-female ratio of 2.4:1, exceeding the ratios reported in comprehensive reviews of RPLs (1.6:1 and 2:1, respectively) (4, 5). The largest tumor diameter reported to date is 19 cm (18), with a median of 12 cm and an average of 12.6 ± 2.4 cm. The most common symptom was dysphagia (76.5%), followed by dyspnea (64.7%), snoring (58.8%), dysphonia (35.3%), and neck swelling (35.3%). Notably, the incidence of each of these symptoms was higher than that reported in comprehensive reviews of RPLs (4, 5), suggesting that symptom frequency and multiplicity tend to increase with tumor size. In the present case, the maximum diameter of 145 mm represents the fourth largest ever reported, and it manifested four of the five aforementioned major symptoms, except for dysphonia.

**Table 1:** The literature reporting giant retropharyngeal lipoma (with a maximum diameter of ≧ 10 cm).

No.	Ref no.	Year	Country	Age	Sex	Size (cm)	Adjective used	Imaging	Management/approach
1	(6)	2005	Lebanon	64	F	12 × 7 × 6	N/A	x-ray, CT	Transcervical
2	(7)	2005	Germany	41	M	12 × 9 × 3	Huge	US, CT, MRI	Transcervical
3	(8)	2006	Iran	60	F	10 × 8 × 4	Huge	CT	Transcervical
4	(9)	2006	Libya	13	M	16.2 × 9.0 × 3.5	Giant	US, CT, MRI	Surgery (not specified)
5	(10)	2006	Poland	40	M	11.7 × 7.2 × 4.5	Huge	CT	Transcervical
6	(11)	2007	India	42	M	11 × 8 × 5	N/A	x-ray, CT	Transoral
7	(12)	2008	Turkey	75	M	12.5 × 8.1 × 2.7	Giant	CT	Observation
8	(13)	2010	India	35	M	11 × 9 × 6	N/A	CT	Transcervical
9	(14)	2011	USA	66	F	10 × 9 × 4.8	Large	CT	Transcervical
10	(15)	2013	Korea	69	F	11 × 10 × 5	N/A	CT, MRI	Transcervical
11	(16)	2015	México	75	F	15	N/A	CT	Transcervical
12	(17)	2015	Indonesia	44	M	19 × 14 × 7	Giant	x-ray, CT	Transcervical
13	(18)	2017	Turkey	45	M	12 × 8 × 4	Giant	CT	Observation
14	(19)	2019	Tunisia	53	M	11.1 × 7.3 × 2.6	Huge	CT	Transcervical
15	(20)	2020	Turkey	24	M	12 × 7	Huge	CT, MRI	Transoral
16	(4)	2022	Saudi Arabia	48	M	13.8 × 10.2 × 4.6	Giant	CT, MRI	Transcervical
17	N/A^a^	2025	Japan	55	M	14.5 × 12 × 4	Giant	CT, MRI	Transcervical

Ref no., reference number; M, male; F, female; N/A, not assigned; CT, computed tomography; MRI, magnetic resonance imaging; US, ultrasound.

a The present case.

**Table 2:** Clinical characteristics of the patients with giant retropharyngeal lipoma (*n* = 17).

Category	Subcategory	*N*	(%)
Age (year)	<40	3	(17.6)
	40–59	8	(47.1)
	≧60	6	(35.3)
	Median (range)	48 (13–75)	
	Average ±SD	50 ± 17	
Sex	Male	12	(70.6)
	Female	5	(29.4)
Maximum diameter (cm)	≦12	11	(64.7)
	>12	6	(35.3)
	Median (range)	12 (10–19)	
	Average ± SD	12.6 ± 2.4	
Symptoms^a^	Dysphagia	13	(76.5)
	Dyspnea	11	(64.7)
	Snoring	10	(58.8)
	Dysphonia	6	(35.3)
	Neck swelling	6	(35.3)
	Obstructive sleep apnea	3	(17.6)
	Stridor	3	(17.6)
	Somnolence	3	(17.6)
Interval from symptom onset to diagnosis	≦12 months	9	(52.9)
	>12 months	5	(29.4)
	Not stated	3	(17.6)
Imaging for diagnosis^a^	CT	17	(100.0)
	MRI	6	(35.3)
	x-ray	3	(17.6)
	US	2	(11.8)
Management	Surgery	15	(88.2)
	Observation	2	(11.8)
Surgical approach^b^	Transcervical	12	(80.0)
	Transoral	2	(13.3)
	Not specified	1	(6.7)
Tracheostomy^b^	Yes	6	(40.0)
	No	9	(60.0)
Tube feeding^b^	Yes^c^	3	(20.0)
	No	12	(80.0)
Outcome^b^	Recovery	12	(80.0)
	Not stated	3	(20.0)
Recurrence^b^	No	11	(73.3)
	Not stated	4	(26.7)

CT, computed tomography; MRI, magnetic resonance imaging; SD, standard deviation; US, ultrasound.

a Since most cases showed more than one symptom and underwent more than one imaging test, the sum of the number exceeds the total number of cases (*n* = 17), and the sum of the percentages exceeds 100.

b *n* = 15.

c One case with percutaneous gastrostomy nutrition was included.

As for imaging, CT was performed in all cases (100%), whereas MRI was combined in 35.3% of the cases. Although both modalities are considered the gold standard for the imaging diagnosis of RPL, CT has been the most widely used as the de facto standard.

As regards treatment, surgical resection was performed in 15 patients (88.2%), while two (11.8%) were managed with observation alone (13, 19) (Table 2). The transcervical approach was most frequently employed (80%), whereas the transoral approach was only applied in two cases (12, 21) (13.3%). In contrast, a previous comprehensive review of RPLs, wherein less than one-third (31.6%) of cases had a maximum diameter of 10 cm or larger, reported a much higher proportion (23 of 73 cases, 34.8%) of transoral approaches (4). This difference suggests that the transoral approach becomes technically more challenging as the tumor increases in size with lateral and caudal expansion, thereby supporting the preferred use of the transcervical approach for giant RPLs. Among the two cases in which a transoral approach was employed, one tumor exhibited marked vertical extension while remaining largely confined to the midline of the retropharyngeal space (12). The other tumor appeared divided into two distinct parts, connected only by a narrow constriction (21), suggesting that it may not necessarily qualify as a “giant” RPL. Conceivably, even in cases of relatively larger RPL, if the tumor remains confined within the midline at a relatively higher level in the pharynx because of its predominantly vertical expansion with minimal lateral and caudal spread, the transoral approach could be a feasible option.

Aside from giant RPLs, among relatively small cases treated with a transoral approach, transoral robotic surgery (TORS) was also reported in a case of RPL measuring 44 × 23 × 14 mm with favorable postoperative results (22). Although the primary indication for TORS has been oropharyngeal cancer, its technical advantages over open surgeries and clinical benefits have also been demonstrated for retropharyngeal lesions (23–25), as well as parapharyngeal tumors (26, 27). However, to achieve precise resection of larger lesions involving the PPS, a strategy combining multiple surgical approaches is reasonable for overcoming the difficulties in surgical access due to its anatomical complexity (28). In a recent systematic review of TORS for parapharyngeal lesions, Chiari et al. compared the application and outcomes between TORS-only versus TORS combined with a trans-cervical approach, outlining the limitations of the former and providing recommendations for each approach (29). In the future, even for giant RPL cases where a trans-cervical approach is essential, combining it with TORS may prove to be beneficial.

If the RPL extends caudally beyond the cricothyroid ligament, as in the present case, exposure of the adjacent RLN is anticipated. Although intraoperative NIM has not been previously reported in RPL surgery, we applied it following the same manner as in conventional thyroid surgery. Even though direct exposure of the RLN was eventually avoided, NIM was considered helpful in this case as an adjunct for locating the RLN during dissection procedure, particularly on the left side where the tumor and dorsal surface of the thyroid were firmly adherent. A case has been reported of a giant RPL with caudal extension, in which transient postoperative hoarseness lasted for three months despite intraoperative preservation of the RLN (17). Given these observations, in RPL surgery involving deep caudal extension of the lesion with an increased risk of RLN injury, the use of NIM may be worth considering to ensure the nerve preservation.

Regarding airway management, tracheostomy was performed in six of the 15 patients (40%) who underwent surgical resection of the giant RPL (Table 2). As expected, this tracheostomy rate was higher than that reported in a comprehensive review of RPLs (21 out of 73 cases, 28.8%) (4). In the absence of clear published guidelines, the decision on whether to perform prophylactic tracheostomy during surgery involving extensive circumferential dissection around the trachea and esophagus is left to the surgeon's discretion. For instance, in cases of the two largest RPLs with maximum diameters of 19 cm and 16.2 cm, given the anticipated difficulty in endotracheal intubation for general anesthesia and postoperative laryngopharyngeal edema, prophylactic tracheostomy was performed (10, 18). Conversely, in the case of the third largest RPL with a maximum diameter of 15 cm, even without preventive tracheostomy, favorable outcomes without any perioperative complications have been reported (17). In our case, although preoperative endoscopy confirmed sufficient glottic exposure with little possibility of difficulty in endotracheal intubation, a prophylactic tracheostomy was performed considering the critical risk of postoperative airway compromise associated with laryngopharyngeal edema.

As for postoperative nutrition, tube feeding was administered in three of 15 patients (20%) who underwent surgery for giant RPL (12, 15, 21) (Table 2). One patient had to undergo percutaneous endoscopic gastrostomy (PEG) preoperatively because of deteriorating dysphagia, which resolved favorably after surgery, allowing successful removal of the PEG tube (15). The rate of tube feeding in patients with giant RPL was also higher than that reported in a comprehensive review of RPLs (nine of 73 cases, 12.3%) (4). According to the European Society for Clinical Nutrition and Metabolism guidelines, although these are primarily intended for patients in the acute phase of stroke, nasogastric tube feeding is recommended when oral intake is expected to be impaired for >7 days, and PEG is recommended if enteral feeding is required for >28 days (30). In the present case, although oral intake was resumed with a liquid diet on POD 2, it took two weeks for the patient to return to a normal diet. Since larger RPLs may enhance the degree of postoperative pharyngeal edema, potentially delaying recovery of normal oral intake, enteral nutrition may serve as a useful alternative when necessary.

Concerning the outcomes of surgery for giant RPLs, resolution of all preoperative symptoms was reported across most cases (12 of 15, 80.0%), while outcome details were not provided for the remaining three cases (20%) (Table 2). Although the duration of postoperative follow-up has been variable and may be insufficient, largely due to the benign nature of the disease, no recurrence has been reported to date, indicating that surgical resection can yield favorable outcomes even in giant RPLs.

As a limitation of this report, due to the extreme rarity of the disease, articles on giant RPL available for the literature review were scarce. Consequently, the review subjects consisted of a small number of previously reported individual cases with heterogeneous clinical data, which inevitably led to information bias such as reporting and observer bias, in addition to inherent selection bias. Moreover, statistical comparison tests could not be applied for the same reason. Further studies based on a larger number of cases are warranted to elucidate the optimal stratified management strategy for giant RPL cases.

## 4 Conclusions

We encountered a case of a giant RPL presenting with various compressive symptoms, all of which fully resolved following surgical removal, highlighting the need to recognize RPL as one of the differential diagnoses in patients exhibiting gradually worsening dysphagia, as well as the efficacy of its surgical treatment. A literature review of giant RPLs suggested an even higher predominance in males, a greater multiplicity of symptoms, and a higher preference for the transcervical approach. Intraoperative use of NIM may serve as a helpful adjunct for preserving the RLN. Furthermore, the necessity of prophylactic tracheostomy and postoperative tube feeding should be assessed on an individual basis.

## Data Availability

The original contributions presented in the study are included in the article/Supplementary Material, further inquiries can be directed to the corresponding author.

## References

[1] Pacifici A Caporro G Pacifici L. Lipomas of the head-neck: a systematic review of literature over the last 10 years. Ann Stomatol (Roma). (2025) 16:263–92. 10.59987/ads/2025.3.263-292

[2] Barnes L. “Tumors and tumor like lesions of the head and neck”. In: Barnes L, editor. Surgical Pathology of the Head and Neck. New York: Marcel Decker Inc (1985) P. 747–58.

[3] Som PM Scherl MP Rao VM Biller HF. Rare presentations of ordinary lipomas of the head and neck: a review. AJNR Am J Neuroradiol. (1986) 7:657–64. 3088944 PMC8334668

[4] Chrysovitsiotis G Sofokleous V Kyrodimos E Papanikolaou V Giotakis E. Retropharyngeal space lipomas. A systematic review of the reported cases in the literature. Indian J Otolaryngol Head Neck Surg. (2022) 74:5630–8. 10.1007/s12070-021-02962-6 36742695 PMC9895578

[5] Alnami RA Saabi SM Mossery RA Alnami BA Al Ghadeeb M. A giant retropharyngeal lipoma: a case report and review of literature. Cureus. (2022) 14:e29022. 10.7759/cureus.29022 36249630 PMC9550210

[6] Muhib M Abidi SLF Ahmed U Afzal A Farooqui A Jamil K. Use of radiologic imaging to differentiate lipoma from atypical lipomatous tumor/well-differentiated liposarcoma: systematic review. SAGE Open Med. (2024) 12:20503121241293496. 10.1177/20503121241293496 39526094 PMC11549689

[7] Haddad FS Zaytoun G Haddad FF. Retropharyngeal lipoma, a benign yet potentially lethal condition - case presentation and review of the literature. Neurosurg Q. (2005) 15:145–54. 10.1097/01.wnq.0000179809.85219.b8

[8] Lohnstein PU Maier W Birkenhager R Schipper J. [The interesting case – case no. 67]. Laryngorhinootologie. (2005) 84:51–3. 10.1055/s-2004-826066 15647978

[9] Behnoud F Hashemian F. A case report of huge retropharyngeal lipoma. Iran J Otorhinolaryngol. (2006) 18:143–5.

[10] El Fortia M El Fagieh M Khalil M Elhamroush H Eldergash O El Gatit A. A massive retropharyngeal lipoma. Eur J Radiol Extra. (2006) 57:9–12. 10.1016/j.ejrex.2005.10.005

[11] Namyslowski G Scierski W Misiolek M Urbaniec N Lange D. Huge retropharyngeal lipoma causing obstructive sleep apnea: a case report. Eur Arch Otorhinolaryngol. (2006) 263:738–40. 10.1007/s00405-006-0050-x 16673079

[12] Radhakrishna Pillai OS Vijayalakshmi S Adarsha TV Thahir M Gopinathan UK Mohammed N. Retropharyngeal lipoma - a case report. Indian J Otolaryngol Head Neck Surg. (2007) 59:360–2. 10.1007/s12070-007-0102-6 23120474 PMC3452255

[13] Lakadamyali H Ergun T Lakadamyali H Avci S. A giant retropharyngeal lipoma showing no change in clinical presentation and size within a two-year follow-up: a case report. Kulak Burun Bogaz Ihtis Derg. (2008) 18:374–6. 19293628

[14] Sethi S Arora V. Use of glidescope and external manipulation in airway management of an unusual retropharyngeal lipoma. J Anaesthesiol Clin Pharmacol. (2010) 26:557–8. 10.4103/0970-9185.74615 21547195 PMC3087252

[15] Chhetri DK. Benign and malignant lipogenic tumors of the retropharyngeal space. Nepalese J ENT Head Neck Surg. (2011) 2:22–4. 10.3126/njenthns.v2i1.6781

[16] Lee HK Hwang SB Chung GH Hong KH Jang KY. Retropharyngeal spindle cell/pleomorphic lipoma. Korean J Radiol. (2013) 14:493–6. 10.3348/kjr.2013.14.3.493 23690719 PMC3655306

[17] López AP Ramírez HJ Ciriaco SV Salvador JMM López NC Maldonado MM. Lipoma retrofaríngeo causante de apnea del sueno y disfagia. Gaceta Mexicana de Oncología. (2015) 14:112–6. 10.1016/j.gamo.2015.07.001

[18] Romdhoni AC Hutahaean F Ahwil C Fadillah IH. Giant retropharyngeal lipoma. Inter J Otorhinolaryngology. (2015) 2:4.

[19] Dilek O Kaya O Yilmaz C Soker G Gulek B Akin MA. A rare cause of obstructive sleep apnea syndrome: retropharyngeal lipoma. Case Rep Radiol. (2017) 2017:2134362. 10.1155/2017/2134362 28912996 PMC5585579

[20] Ghammam M Houas J Bellakhdher M Abdelkefi M. A huge retropharyngeal lipoma: a rare cause of dysphagia: a case report and literature review. Pan Afr Med J. (2019) 33:12. 10.11604/pamj.2019.33.12.18541 31303957 PMC6607450

[21] Aydin U Karakoc O Binar M Arslan F Gerek M. Intraoral excision of a huge retropharyngeal lipoma causing dysphagia and obstructive sleep apnea. Braz J Otorhinolaryngol. (2020) 86 Suppl 1:8–10. 10.1016/j.bjorl.2016.10.011 27939855 PMC9422589

[22] Heaton CM Ahmed SR Ryan WR. Transoral robotic surgery (TORS) for excision of a retropharyngeal intramuscular lipoma. Auris Nasus Larynx. (2017) 44:742–4. 10.1016/j.anl.2016.10.010 27956103

[23] Garas G Roland NJ Lancaster J Zammit M Manon VA Davies K. Novel strategies for managing retropharyngeal lymph node metastases in head and neck and thyroid cancer with transoral robotic surgery (TORS). Ann Surg Oncol. (2022) 29:7881–90. 10.1245/s10434-022-12208-6 35842533

[24] Givi B Troob SH Stott W Cordeiro T Andersen PE Gross ND. Transoral robotic retropharyngeal node dissection. Head Neck. (2016) 38 Suppl 1:E981–6. 10.1002/hed.24140 26040490

[25] Ding X Lin QG Zou X Liu YP Hua YJ Xie YL. Transoral robotic retropharyngeal lymph node dissection in nasopharyngeal carcinoma with retropharyngeal lymph node recurrence. Laryngoscope. (2021) 131:E1895–E902. 10.1002/lary.29319 33378575

[26] De Virgilio A Costantino A Mercante G Di Maio P Iocca O Spriano G. Trans-oral robotic surgery in the management of parapharyngeal space tumors: a systematic review. Oral Oncol. (2020) 103:104581. 10.1016/j.oraloncology.2020.104581 32058293

[27] Larson AR Ryan WR. Transoral excision of parapharyngeal space tumors. Otolaryngol Clin North Am. (2021) 54:531–41. 10.1016/j.otc.2021.03.001 34024481

[28] Ferrari M Schreiber A Mattavelli D Lombardi D Rampinelli V Doglietto F. Surgical anatomy of the parapharyngeal space: multiperspective, quantification-based study. Head Neck. (2019) 41:642–56. 10.1002/hed.25378 30592348

[29] Chiari F Filippini DM Serafini E Fermi M Presutti L Maniaci A. Transoral robotic approaches for benign and malignant parapharyngeal space tumors: comparative analysis and systematic review. Auris Nasus Larynx. (2025) 52:776–84. 10.1016/j.anl.2025.10.008 41183427

[30] Burgos R Breton I Cereda E Desport JC Dziewas R Genton L. ESPEN guideline clinical nutrition in neurology. Clin Nutr. (2018) 37:354–96. 10.1016/j.clnu.2017.09.003 29274834

